# The Effects of *Bacillus licheniformis* on the Growth, Biofilm, Motility and Quorum Sensing of *Salmonella typhimurium*

**DOI:** 10.3390/microorganisms13071540

**Published:** 2025-06-30

**Authors:** Wenwen Peng, Haocheng Xu, Meiting Zhang, Baoyang Xu, Bing Dai, Caimei Yang

**Affiliations:** College of Animal Science and Technology, College of Veterinary Medicine, Zhejiang A&F University, Hangzhou 311300, China; pwenwen2022@163.com (W.P.); haochengxu03@foxmail.com (H.X.); mittyours@163.com (M.Z.); baoyangxu@126.com (B.X.); daibing2008@126.com (B.D.)

**Keywords:** *Bacillus licheniformis*, *Salmonella typhimurium*, biofilm

## Abstract

With 80% of bacterial infections occurring as biofilms, biofilm-related infections have evolved into a critical public health concern. Probiotics such as *Bacillus licheniformis* have emerged as promising alternatives, offering new avenues for effective treatment. This study aimed to evaluate the activity of *licheniformis* against the growth, biofilm formation, motility, and quorum sensing (QS) of *Salmonella typhimurium*. Several experiments were conducted: The minimum inhibitory concentration (MIC) of *Bacillus licheniformis* against *Salmonella typhimurium* was determined to be 0.5 mg/mL using the broth microdilution method. The inhibition zone of 100 mg/mL of *B. licheniformis* against *Salmonella typhimurium* was 19.98 ± 1.38 mm; the time-growth curve showed that *B. licheniformis* can effectively inhibit the growth of *Salmonella typhimurium*. In biofilm experiments, at the MIC of *B. licheniformis*, the inhibition rate of immature biofilm of *Salmonella typhimurium* was 86.9%, and it significantly reduced the production of biofilm components (EPS, e-DNA, and extracellular proteases) (*p* < 0.05). The disruption rate of mature biofilm by *B. licheniformis* at the MIC was 66.89%, and it significantly decreased the levels of biofilm components (EPS and e-DNA) (*p* < 0.5). Microscopic observation showed that both the MIC and 1/2 MIC of *B. licheniformis* could reduce the number of bacteria in the *Salmonella typhimurium* biofilm, which was not conducive to the formation and maintenance of the biofilm structure. Swimming/Swarming assays and QS experiments confirmed that *B. licheniformis* inhibits the motility of *Salmonella typhimurium* and the secretion of AI-1-type quorum sensing molecules and downregulates the AI-2 quorum sensing system by upregulating *lsr* gene expression. These findings suggest that *B. licheniformis* could be a potential antimicrobial agent and biofilm inhibitor.

## 1. Introduction

*Salmonellosis* is particularly a serious problem in the livestock and poultry industry. It is widely distributed in the environment, domestic animals, and wild animals. Transmitted through the fecal-oral route via contaminated food or water, it is an important zoonotic pathogen responsible for gastrointestinal infections and can lead to the death of humans or domestic animals [1,2]. Studies have found that 50% of *Salmonella* strains isolated from poultry farms were capable of producing biofilms [3]. Biofilms help *Salmonella* survive in hostile environments. *Salmonella* can form biofilms on produced food products, as well as in processing areas on poultry farms, such as walls, floors, pipes, and sewers, and on contact surfaces, such as stainless steel, aluminum, nylon, rubber, plastic, polystyrene, and glass [4,5,6].

Biofilms enhance bacterial drug resistance through mechanisms such as impeding the penetration of antibiotics, altering the local environment within the biofilms, and inducing bacterial dormancy [7]. It can be challenging to remove *Salmonella* biofilms from poultry environments [8]. Disinfection is not effective against bacteria; some studies showed that the detection rate of *Salmonella* in poultry environmental samples after cleaning and disinfection of broiler and laying hen houses is higher [9,10].

Studies have shown that probiotics kill or hinder pathogenic bacteria by producing substances such as bacteriocins, hydrogen peroxide, and organic acids [11]. Common single probiotics include *Lactic Acid Bacteria*, *Bacillus*, *yeast*, etc. [12]. As a representative, *Bacillus licheniformis* inhibits the growth and reproduction of pathogens by producing antibacterial substances such as lichenysin and bacitracin and plays a positive role in promoting the digestion and absorption of nutrients, and it is widely used in the livestock industry as a growth promoter and a competitive exclusion agent [13,14]. Recent research highlights its anti-biofilm potential: Strain V9T14s lipopeptide inhibits biofilm formation in *E. coli* and *S. aureus* [15]; *Bacillus tropicalis* extracellular protein D1 prevents biofilm adhesion in *C. albicans*, *P. aeruginosa*, and *Bacillus* Pontospira while dispersing preformed biofilms [16]; and Indian prawn-isolated DAHB1 reduces intestinal vibrios by quenching quorum sensing to disrupt biofilm development [17]. These findings confirm *B. licheniformis*’s antibacterial and anti-biofilm activities, but its effects and mechanisms against *Salmonella typhimurium* biofilms remain unclear, particularly in quorum sensing regulation and biofilm component degradation pathways.

This study fills the existing knowledge gap on *B. licheniformis* against *Salmonella typhimurium* biofilms and evaluates the potential of *B. licheniformis* as an antibacterial agent and an anti-biofilm inhibitor. The objectives of this work are (1) to investigate the inhibitory ability of *B. licheniformis* on the growth and biofilm yield of *Salmonella typhimurium*, (2) to investigate the effects of *B. licheniformis* on the composition and structure of mature and immature biofilms of *Salmonella typhimurium*, and (3) to investigate the effects of *B. licheniformis* on *Salmonella typhimurium* motility and quorum sensing. This study clarifies the multiple roles of *B. licheniformis* in inhibiting the growth of *Salmonella typhimurium*, resisting biofilm formation, restricting motility, and interfering with quorum sensing. It provides an experimental basis for combating *Salmonella typhimurium* infections and addressing related public health issues, as well as lays a theoretical foundation for the development of novel antibacterial and anti-biofilm inhibitors based on *B. licheniformis*.

## 2. Materials and Methods

### 2.1. Materials, Bacteria Strains, and Reagents

The *Bacillus licheniformis 9385* (*Bacillus licheniformis 9385*, BL 9385) used in this study was sourced from Zhejiang Huijia Biotechnology Co., Ltd. (Anji, China), and *Salmonella typhimurium* (*Salmonella typhimurium*, CMCC 50115, ST) was purchased from the China General Microbiological Culture Collection Center. The *Chromobacterium violaceum* (*Chromobacterium violaceum*, CV ATCC 12472) was kindly provided by Professor Sun Wuwen from the College of Animal Science and Technology of Jilin Agricultural University.

Luria-Bertani Broth Medium Formula and Its Manufacturing Companies: Sodium Chloride, Sinopharm Group Shanghai Chemical Reagent Co., Ltd. (Shanghai, China); Tryptone, Beijing Aoboxing Biotechnology Co., Ltd. (Beijing, China); Yeast Extract, Hangzhou Best Biotechnology Co., Ltd. (Hangzhou, China); Agar Powder, Changsha Dingguo Changsheng Biotechnology Co., Ltd. (Changsha, China); DMSO and EDTA, Tianjin Yongda Chemical Reagent Co., Ltd. (Tianjin, China); Crystal Violet, Sangon Biotech (Shanghai) Co., Ltd. (Shanghai, China); Absolute ethanol, Sangon Biotech (Shanghai) Co., Ltd. (Shanghai, China); PBS, Wuhan Servicebio Technology Co., Ltd. (Wuhan, China); 5% phenol solution, Yishijiu (Lianyungang, China) Biotechnology Co., Ltd.; Anti-fluorescence attenuation sealant, Beijing Solarbio Science and Technology Co., Ltd. (Beijing, China); Neomycin sulfate and Propidium Iodide (PI), Shanghai Aladdin Biochemical Technology Co., Ltd. (Shanghai, China); MolPure Bacterial DNA kit, Yeasen Biotechnology (Shanghai) Co., Ltd. (Shanghai, China).

### 2.2. Determination of the MIC

The MIC (the Minimum Inhibitory Concentration) of *B. licheniformis* against *Salmonella typhimurium* was determined as described by Li et al. (2024) [18]. *Bacillus licheniformis* was dissolved in sterile LB medium and then filtered into a sterile test tube using a 0.22 μm filter. Next, two-fold serial dilutions (100 μL) of the above sterile LB medium were performed in a 96-well microtiter plate, with the final concentration ranging from 0.008 to 2.000 mg/mL. The wells were grouped as follows: A1 served as the blank control group, A2 as the ST experimental group, A3 as the NS and ST co-treatment group, and A4–A12 as BL and ST co-treatment groups. Each well was inoculated with 100 μL of the bacterial suspension (OD_600nm_ = 0.1, 5 × 10^8^ CFU/mL). After incubating at 37 °C with continuous shaking for 16 h, the cell growth was measured by determining the absorbance at OD_600nm_ using a Multiskan FC multifunctional microplate reader (Thermo Fisher Scientific Inc., Waltham, MA, USA) This experiment was repeated three times.

### 2.3. Determination of the DIZ

The method was adapted from the agar diffusion technique reported earlier, with several adjustments made [19,20]. First, the ST solution (OD_600nm_ = 0.1) is evenly spread on the blank LB medium. Then, blank filter paper discs (7 mm) were placed on the surface of the agar of the above-mentioned LB medium, and 20 μL of different concentrations of *B. licheniformis* were added onto the filter paper discs. After that, the setup was placed in an incubator at a constant temperature of 37 °C and cultured in an inverted position for 16 h. The diameter of the inhibition zone (DIZ) was measured using a G1 Automatic Colony Counter (Xunshu Technology Co., Ltd., Hangzhou, China) so that the antibacterial potency of *B. licheniformis* could be represented. This experiment was repeated three times.

### 2.4. Growth Curve Assay

The antibacterial capacity of *B. licheniformis* against *Salmonella typhimurium* was gauged by measuring the growth curve of *Salmonella typhimurium*. And the method, which was slightly modified, was based on the protocol proposed by Wang et al. (2020) [19]. *B. licheniformis* was added to the sterile LB medium and was serially diluted to reach concentrations of 1/16 MIC, 1/8 MIC, 1/4 MIC, 1/2 MIC, and MIC, respectively. Then, the solutions were filtered through 0.22 μm filters into new sterile test tubes. The sterile LB medium was used as a control. The *Salmonella typhimurium* bacterial solution was inoculated into the above-mentioned LB medium. All the LB media were placed in an incubator at 37 °C, 140 rpm for 24 h. The growth of *Salmonella typhimurium* was recorded by measuring the OD_600nm_ every 2 h.

### 2.5. Preparation of Biofilm

The cultivation experiment of biofilm was divided into six groups: The ST group, the MIC solution + ST group, the 1/2 MIC solution + ST group, the 1/4 MIC solution + ST group, the 1/8 MIC solution + ST group, and the 1/16 MIC solution + ST group. According to the grouping, *B. licheniformis* was added to the sterile LB medium, and culture media with different MIC concentrations were prepared.

#### 2.5.1. Preparation of Immature Biofilms

A static biofilm formation assay was performed in 24-well polystyrene plates. The different MIC concentrations of *B. licheniformis* culture medium were added to 24-well polystyrene plates, and 20 μL of ST (10^7^ CFU/mL, OD_600nm_ = 0.1) bacterial solution was added to each well. Finally, the 24-well polystyrene plates were incubated for 24 h without shaking at 37 °C.

#### 2.5.2. Preparation of Mature Biofilm

The sterile LB medium was added to 24-well polystyrene plates, and 20 μL of ST (10^7^ CFU/mL, OD_600nm_ = 0.1) bacterial solution was added to each well. The 24-well polystyrene plates were incubated for 24 h without shaking at 37 °C. Then, the culture solution was discarded. Subsequently, the different MIC concentrations of *B. licheniformis* culture medium were added to 24-well polystyrene plates, and the plates were further cultivated for 24 h without shaking at 37 °C.

### 2.6. Crystal Violet Biofilm Assay

After the biofilm was cultivated as described in Section 2.5, the culture solution was discarded. The biofilm was then washed three times with PBS, and 0.1% crystal violet was added to each well. After 20 min at room temperature, the microplate was emptied and washed three times with PBS. Finally, 95% anhydrous ethanol was added to solubilize the stained biofilm cells, and the absorbance of the solution was measured at 545 nm to quantify the total biofilm biomass.

The inhibition equation of biofilm is

$$\mathrm{Biofilm}\;\mathrm{inhibition}/\mathrm{damage}\;\mathrm{rate}(\%)=\frac{{OD}_{ST}-{OD}_{BL}}{{OD}_{ST}-{OD}_{CON}}\times 100\%$$

*OD_ST_*: The OD value of the *Salmonella typhimurium* group; *OD_BL_*: The OD value of the *B. licheniformis* (BL) treatment group; *OD_CON_*: The OD value of the CON group.

### 2.7. Biofilm Components

#### 2.7.1. Exopolysaccharides Quantification Assay

The concentrated sulfuric acid-phenol method was used to determine the Exopolysaccharides (EPS) content of biofilms. To the biofilm prepared in Section 2.5, 500 μL of PBS was added to each well. Then, 500 μL of 5% phenol solution was added to each well, followed by the immediate addition of 2500 μL of concentrated sulfuric acid. The mixture was incubated at 37 °C for 1 h. The OD_490_ was measured, and the calculation formula was the same as that in the Crystal violet biofilm assay described in Section 2.6.

#### 2.7.2. Extracellular DNA (e-DNA) Quantification Assay

After the biofilm was cultivated as described in Section 2.5, the culture solution was discarded. The biofilm was washed three times with PBS, and then 5 µL of EDTA (0.5 M) was added to each well. The plate was left to stand at 4 °C for 1 h, after which 500 µL of TEN (Tris-EDTA-NaCl) buffer was added to each well to resuspend the biofilm cells. The suspension was centrifuged at 18,000× *g* for 5 min at 4 °C, and 500 µL of the supernatant was transferred to an EP tube containing 1500 µL of TE (Tris-EDTA) buffer. The mixture and an equal volume of binding buffer (MolPure Bacterial DNA kit) were then added to the adsorption column. After standing at room temperature for 2 min, the column was centrifuged at 18,000× *g* for 60 s at 4 °C. The adsorption column was washed with washing buffer, followed by centrifugation at 18,000× *g* for 30 s. Finally, sterile water was added, and centrifugation was performed to collect e-DNA. The concentration of e-DNA was measured using a NanoDrop spectrophotometer (Allsheng, Hangzhou, China).

#### 2.7.3. Protease Quantification Assay

The agar diffusion method was used to detect the content of proteases in biofilms. According to the experimental method in Section 2.5, ST biofilm was cultivated in the presence of *B. licheniformis*, and no glass slides were placed in the 24-well plate. Approximately 50 mL of distilled water was mixed with 4 g of skim milk powder, heated and dissolved in a 60 °C water bath, magnetically stirred for 30 min, and separately sterilized at 115 °C for 10 min. For preparing LB agar medium, 50 mL less water was added, and the medium was sterilized at 121 °C for 15 min. A small amount of agar medium was poured into the bottom of a petri dish, and a high-pressure sterilized Oxford cup was placed on the plate. Skim milk was mixed evenly with LB solid medium, poured into the petri dish, and after solidification, the Oxford cup was removed. Two hundred microliters of the bacterial solution prepared in Section 2.5 was added to each well, and the plate was incubated at 30 °C for 24–48 h. The diameter of the transparent ring was recorded using a G1 Automatic Colony Counter (Xunshu Technology Co., Ltd., Hangzhou, China).

### 2.8. Microscopic Observation

A 10 mm sterile cell crawl sheet was added to each well of a sterile 24-well polystyrene plate, and the biofilm was further cultured as described in Section 2.5.

#### 2.8.1. Optical Microscope Assay

The crystal violet staining observation was carried out using the experimental method of Li et al. (2024) [18]. The biofilm-climbing coating was taken, and the surface planktonic bacteria were washed away with PBS to remove non-adherent cells. Then, they were stained with 1% (*w*/*v*) crystal violet solution for 30 min, and the excess crystal violet was washed away with sterile PBS. After that, they were observed and photographed through an optical microscope (BDS400, CNOPTEC Co., Ltd., Hangzhou, China).

#### 2.8.2. Fluorescence Microscopy Assay

According to the experimental method of Tao et al. (2025) [21], the effect of *B. licheniformis* on bacterial membrane integrity was analyzed using a fluorescence confocal microscope, with some modifications. First of all, the biofilm cover slips cultured in Section 2.5 were taken out, washed with PBS to remove planktonic bacteria, and transferred to a new 24-well plate. Subsequently, 100 μL of PI staining solution was added, and the plate was incubated at 37 °C in the dark for 15 min. After slide preparation, a drop of anti-fluorescence quenching agent was applied to a glass slide. The stained cell climbing slide was then placed upside down on the glass slide and fixed using a mounting medium. Finally, the biofilm was observed and photographed using an Olympus laser confocal microscope (BX61W1-FV1000, Olympus, Tokyo, Japan).

#### 2.8.3. Scanning Electron Microscope (SEM) Assay

The SEM experiment was conducted following the experimental method of Qin et al. (2024) [22]. The biofilm climbing slides prepared in Section 2.5 were taken, and the surface planktonic bacteria were washed away with PBS. Then, the samples were fixed overnight with a 2.5% glutaraldehyde solution. Subsequently, they were dehydrated using a graded ethanol series (30%, 50%, 70%, 80%, 90%, and 100% ethanol). After freeze-drying, the cell-climbing slides were sputter-coated with gold, and finally, they were observed and photographed using a scanning electron microscope (SU8010, Hitachi High-Tech Corporation, Tokyo, Japan).

### 2.9. Motility Assay

Swimming and swarming motility of *Salmonella typhimurium* was assayed according to previous methods with few modifications [18]. An LB medium containing 0.3% agar was deposited into a plate for the swimming assay. *B. licheniformis* at the various concentrations of MIC and an equal volume of LB medium were added to separate wells as treatment and control, respectively. Approximately 5 μL of culture of ST was then punctured at the center of each plate; after incubation at 37 °C for 8 h, bacterial migration was recorded from the center to the edge of the plates. For the swarming test, it was conducted by adding 5 μL of ST culture to the center of a six-well plate containing LB medium with 0.6% agar. The plates were incubated at 37 °C for 6 h, and the radius of migration was measured.

### 2.10. Assay for AI-1 Activity of ST

According to the method of Peng et al. (2018) [23], a quantitative inhibition experiment of purple pigment was conducted, and some modifications were made. Specifically, the expanded CV ATCC 12472 bacterial solution was first diluted to OD_600nm_ = 0.1. Take sterile glass test tubes and add 10 mL of the above ATCC 12472 bacterial solution. Different doses of *Bacillus licheniformis* are added to each group to adjust the concentration to MIC, 1/2 MIC, 1/4 MIC, 1/8 MIC, and 1/16 MIC. Finally, the test tubes were incubated at 30 °C and 140 rpm for 24 h. The culture medium was centrifuged at 5000× *g* and 4 °C for 5 min to precipitate the insoluble purple pigment. The supernatant was discarded, and 0.5 mL DMSO was added. The mixture was vigorously shaken for 2 min to dissolve the purple pigment, followed by centrifugation at 10,000× *g* and 4 °C for 10 min. A 200 μL of the solution was taken, and the absorbance of the soluble purple pigment was measured at 585 nm using a 96-well plate.

### 2.11. Data Analysis Real-Time Polymerase Chain (PCR) Analysis

Following *Bacillus licheniformis* treatment, total RNA extraction from *Salmonella typhimurium* cells utilized RNAiso Plus (TaKaRa, San Jose, CA, USA). cDNA synthesis followed the manufacturer’s protocol, with subsequent amplification via a real-time quantitative PCR system (Bio-Rad, Hercules, CA, USA). Messenger RNA (mRNA) expression was calculated using a specific method and normalized to 16sRNA. Primer sequences appear in Table 1.

### 2.12. Data Analysis

In this experiment, Excel 2016 was employed for data sorting, and GraphPad Prism Software 8.0 was utilized for mapping. The IBM SPSS Statistics 26.0 statistical software was adopted to conduct one-way ANOVA variance analysis, followed by Duncan’s test. Mean ± Standard Error (Mean ± SE). Different lowercase letters indicate significant differences (*p* < 0.05). Finally, PowerPoint was used for composing the group figures.

## 3. Results

### 3.1. Effect of Lipopeptides on Growth of Salmonella typhimurium

The MIC of *B. licheniformis* against *Salmonella typhimurium* is 0.5 mg/mL. The diameters of the DIZ generated by the *B. licheniformis* or NS against ST strains are reported in Table 2. The results show that the mean DIZ values of different concentrations of *B. licheniformis* against *Salmonella typhimurium* are 19.98 mm, 18.31 mm, 16.26 mm, 13.79 mm, and 11.56 mm.

**Figure 1:**
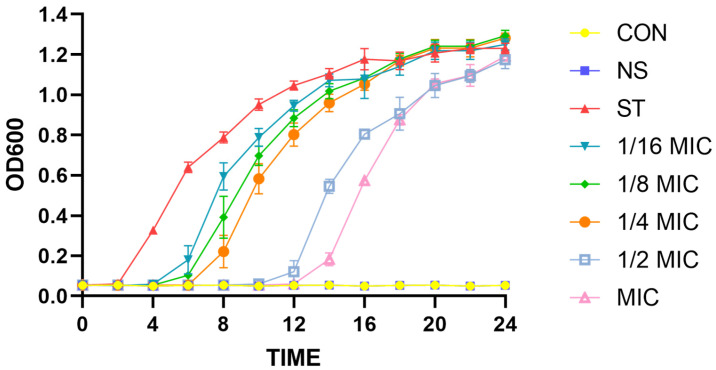
Effect of different treatment dosages of *Bacillus licheniformis* on growth of *Salmonella typhimurium*. CON is blank control group; NS is positive control group, which is neomycin sulfate at a concentration of 50 μg/mL; ST is negative control group; and the 1/16 MIC, 1/8 MIC, 1/4 MIC, 1/2 MIC, and MIC groups are the treatment groups with different concentrations of *B. licheniformis*.

To further verify the antibacterial effect of *B. licheniformis* on ST, the growth curve analysis of ST under different concentrations of *B. licheniformis* is shown in Figure 1. From 0 to 12 h, the MIC (0.5 mg/mL) and 1/2 MIC (0.25 mg/mL) concentrations of *B. licheniformis* significantly inhibit ST growth. However, the growth curve of ST from 12 to 24 h shows that the inhibitory effect of *B. licheniformis* on the growth of planktonic *Salmonella typhimurium* is weakened in the liquid environment, and there is no significant difference at 24 h.

These findings suggest that *B. licheniformis* possesses notable antibacterial properties against ST.

### 3.2. Effect of B. licheniformis on the Quantity of Biofilm of Salmonella typhimurium

**Figure 2:**
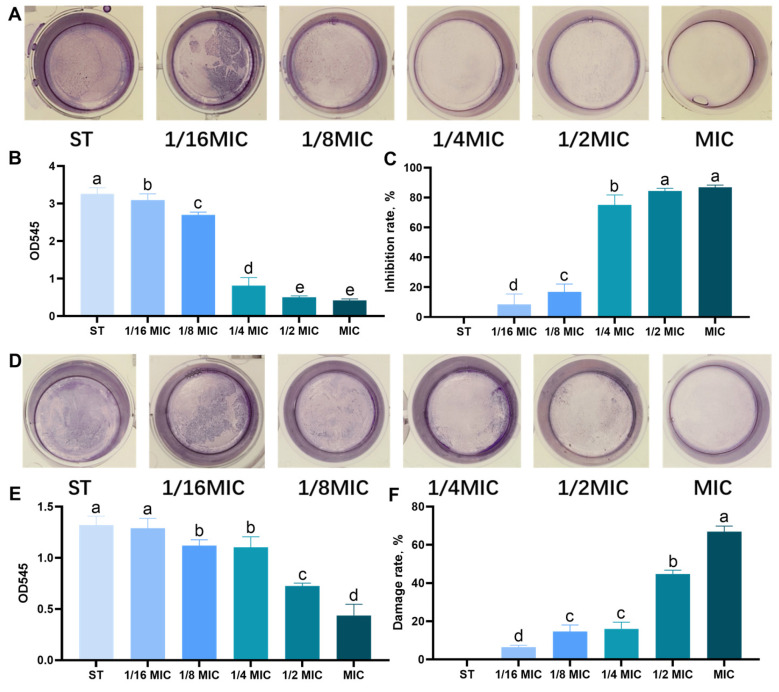
Effect of *B. licheniformis* on quantity of biofilm of *Salmonella typhimurium*. (**A**,**D**) Effects of *B. licheniformis* at different MICs on the immature/mature biofilms of *Salmonella typhimurium*. (**B**,**E**) Detecting the quantity of immature/mature biofilms formed by *Salmonella typhimurium* under crystal violet staining. (**C**,**F**) Inhibition/destruction rate of the biofilms of Salmonella. Different lowercase letters between groups indicate significant differences (*p* < 0.05).Overall, *B. licheniformis* not only inhibits bacterial growth but also effectively impairs biofilm development, highlighting its importance in combating bacterial infections associated with biofilm formation.

The encouraging results obtained through the MIC, DOZ, and growth curve tests prompt us to investigate the potential inhibitory effect of *B. licheniformis* on the biofilm production of the ST considered in this study. The crystal violet biofilm assay was used to evaluate the effect on immature and mature biofilms. *B. licheniformis* was tested at different concentrations of MIC and showed a concentration-dependent trend in its anti-biofilm activity, which was consistent in immature (Figure 2A–C) and mature (Figure 2D–F) biofilms. Notably, even at 1/16 MIC, *B. licheniformis* significantly reduced the content of immature ST biofilms (Figure 2B). As presented in Figure 2C, the average inhibition rates of ST immature biofilm formation in each MIC group were 86.90%, 84.45%, 75.07%, 16.86%, and 8.22%, respectively (*p* < 0.05). Specifically, at 1/8 MIC, *B. licheniformis* effectively disrupted mature ST biofilms and decreased their biomass (*p* < 0.05). As presented in Figure 2F, the average disruption rates of ST mature biofilms within each MIC group were 66.89%, 44.86%, 16.12%, 14.68%, and 6.42%, respectively (*p* < 0.05).

### 3.3. Effect of B. licheniformis on Component of Biofilm of Salmonella typhimurium

#### 3.3.1. Quantification of Exopolysaccharides

**Figure 3:**
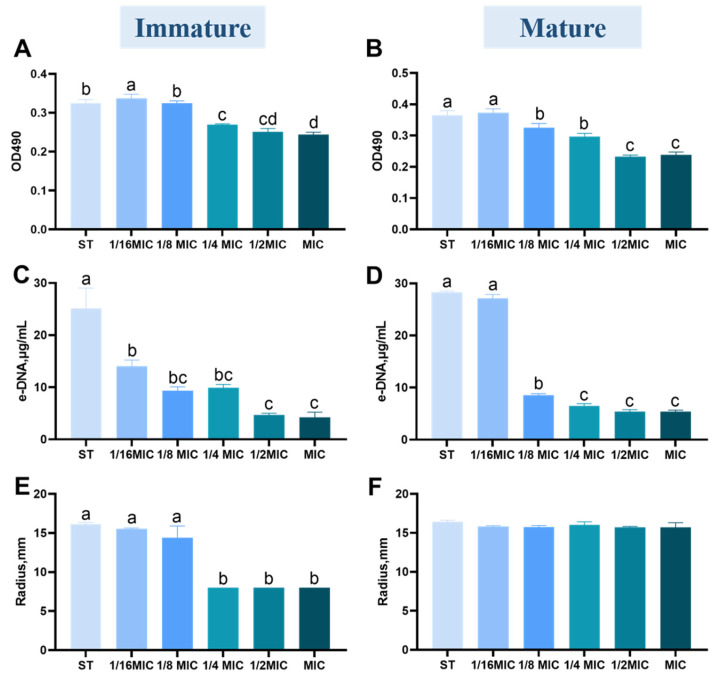
Effect of *B. licheniformis* on components of biofilm of *Salmonella typhimurium*. (**A**,**B**) The content of exopolysaccharides in mature/immature biofilms of *Salmonella typhimurium*; (**C**,**D**) The content of extracellular DNA (e-DNA) in mature/immature biofilms of *Salmonella typhimurium*; (**E**,**F**) The content of extracellular proteases in mature/immature biofilms of *Salmonella typhimurium*. Different lowercase letters between groups indicate significant differences (*p* < 0.05).

Figure 3A,B show the effect of *B. licheniformis* on exopolysaccharides in immature/mature ST biofilm at different MIC concentrations. *B. licheniformis* inhibited the relative content of exopolysaccharides in a concentration-dependent manner.

Notably, exopolysaccharide content decreases significantly at concentrations above 1/4 MIC (*p* < 0.05). The lack of significant difference between the MIC and 1/2 MIC groups indicates that *B. licheniformis* achieves maximal inhibition of exopolysaccharides at 1/2 MIC.

#### 3.3.2. Quantification of e-DNA

e-DNA is present on the bacterial cell surface and enhances adhesion and surface aggregation through acid-base interactions [24]. The results are shown in Figure 3C,D. Once the *B. licheniformis* concentration surpasses 1/16 of the MIC value, it notably restrains the synthesis of e-DNA within the immature biofilm. (*p* < 0.05) However, when the concentration exceeds 1/8 MIC, the content of e-DNA in the *B. licheniformis* group is significantly reduced in the mature biofilm experimental group (*p* < 0.05).

#### 3.3.3. Quantification of Protease

The effect of *B. licheniformis* on ST biofilm proteases is shown in Figure 3E,F. As shown in Figure 3E, when the concentration of *B. licheniformis* exceeds 1/16 MIC, protease synthesis is significantly inhibited in immature biofilms (*p* < 0.05). In contrast, *B. licheniformis* does not significantly affect proteases in mature biofilms (*p* > 0.05).

### 3.4. Effect of B. licheniformis on the Structure of Biofilm of Salmonella typhimurium

#### 3.4.1. Optical Microscope

**Figure 4:**
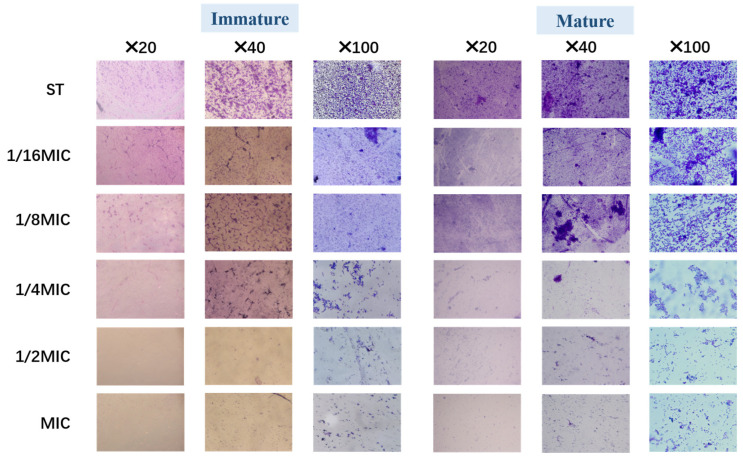
The biofilm of *Salmonella typhimurium* in groups was stained with crystal violet for light field microscopy.

After crystal violet staining, the structure of the biofilm was observed under a light microscope at different multiples. Figure 4 illustrates that both immature and mature *Salmonella typhimurium* biofilm were affected at different *B. licheniformis* concentrations and showed a concentration-dependent trend in its anti-biofilm activity.

As shown in Figure 4, compared with the control (ST group), the bacterial density, matrix, and structural compactness of *Salmonella typhimurium* biofilms decreased and became looser with increasing *B. licheniformis* concentration, especially at concentrations exceeding 1/4 MIC. This result suggests that *B. licheniformis* reduces bacterial numbers in *Salmonella typhimurium* biofilms, exhibiting similar effects on both mature and immature biofilms.

#### 3.4.2. Confocal Fluorescence Microscope

**Figure 5:**
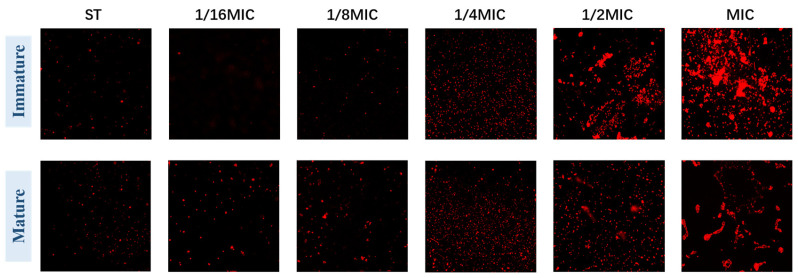
The biofilm of *Salmonella typhimurium* in groups was stained with PI for fluorescence confocal microscopy. (The magnification is 60×).

Immunofluorescence observation further confirmed that *B. licheniformis* can inhibit biofilms. In ST-formed biofilms, most bacteria are dead (stained red). Figure 5 (Immature) shows that with the increase in *B. licheniformis* concentration, the number of dead bacteria in the biofilm gradually increases, indicating that *B. licheniformis* promotes *Salmonella typhimurium* death during co-culture. Meanwhile, mature biofilm groups show results consistent with immature ones (Figure 5 (Mature)).

These images confirm the results from other methodologies and demonstrate that *Salmonella typhimurium* decreases its biofilm-forming ability in the presence of *B. licheniformis*, while *B. licheniformis* disrupts existing biofilms.

#### 3.4.3. Scanning Electron Microscope

**Figure 6:**
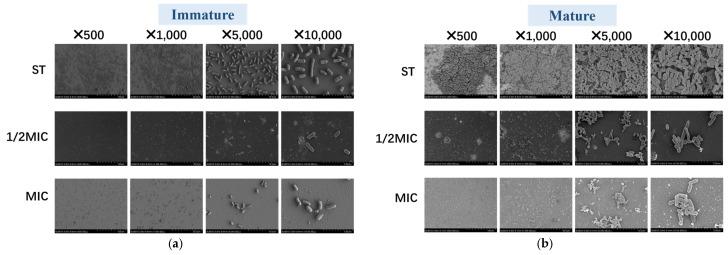
The SEM pictures of biofilm of *Salmonella typhimurium* in groups. (**a**) The SEM pictures of the immature biofilm of *Salmonella typhimurium* in groups; (**b**) The SEM pictures of the mature biofilm of *Salmonella typhimurium* in groups.

To observe the effect of *B. licheniformis* on *Salmonella typhimurium*, bacterial morphology is scanned using SEM. Figure 6 shows that compared with immature biofilms, the ST biofilms in the mature biofilm group thicken, the number of bacteria increases, and the structure becomes more compact as time increases. And bacteria in the ST group are dense, with mature biofilms being even denser. In both immature and mature biofilm assays, bacterial numbers in the 1/2 MIC and MIC groups are significantly lower than those in the ST group. Notably, the bacterial count in the 1/2 MIC immature biofilm is significantly lower than that in the *Salmonella typhimurium* immature biofilm. These results align with the aforementioned experimental findings, further demonstrating the potent antibiofilm activity of *B. licheniformis*, as it can both prevent biofilm formation and disrupt mature biofilms.

### 3.5. Effect of B. licheniformis on Salmonella typhimurium Migration

**Figure 7:**
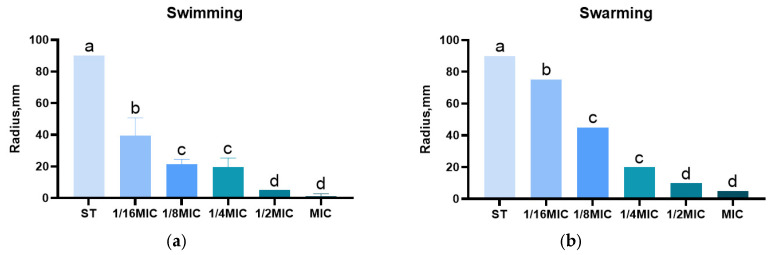
Effect of *B. licheniformis* on *Salmonella typhimurium* migration. (**a**) Effect of different dosage lipopeptide treatment on the level of swimming; (**b**) Effect of different dosage lipopeptide treatment on the level of swarming. Different lowercase letters between groups indicate significant differences (*p* < 0.05).

The swarming and swimming ability of *Salmonella typhimurium* is judged by migration radius. The swimming radii of the groups are 90 mm, 40 mm, 20 mm, 20 mm, 5 mm, and 2 mm, respectively. Similarly, the swarming radii of the groups are 90 mm, 70 mm, 40 mm, 15 mm, 10 mm, and 5 mm, respectively. Furthermore, as shown in Figure 7, the swimming and swarming motility of *Salmonella typhimurium* exposed to *B. licheniformis* at a concentration of 1/16 MIC is significantly suppressed (*p* < 0.001).

### 3.6. Effect of B. licheniformis on AI-1 Activity Produced

**Figure 8:**
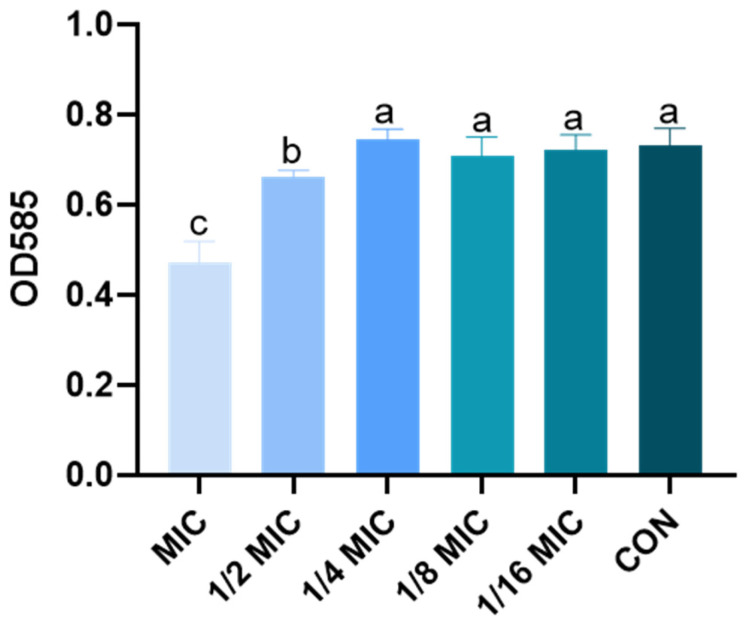
Effect of *B. licheniformis* on AI-1 activity produced. Different lowercase letters between groups indicate significant differences (*p* < 0.05).

To determine whether *B. licheniformis* regulates the quorum sensing of *Salmonella typhimurium*, AI-1 activity produced by ATCC 12472 is examined in the presence or absence of *B. licheniformis*. In the purple pigment experiment, ATCC 12472 is cocultured with different concentrations of *B. licheniformis* medium, and the amount of purpurin produced by ATCC 12472 is significantly reduced in the 1/2 MIC and MIC groups compared with the CON group (Figure 8).

### 3.7. Effect of B. licheniformis on Salmonella typhimurium QS Genes

**Figure 9:**
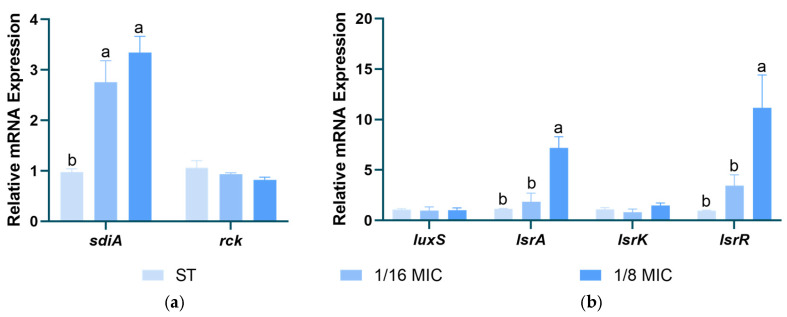
Effect of *B. licheniformis* on QS genes. (**a**) Effect of *B. licheniformis* on QS genes of AI-1; (**b**) Effect of *B. licheniformis* on QS genes of AI-2. Different lowercase letters between groups indicate significant differences (*p* < 0.05).

Figure 9 shows the effects of *B. licheniformis* on the gene expression of *Salmonella typhimurium* AI-1 and AI-2. The results indicate that *B. licheniformis* significantly upregulates the expression of the *sdiA* gene in the type I QS system and the *lsrA* and *lsrR* genes in the type II QS system, thereby inhibiting the type II QS system (*p* < 0.05).

## 4. Discussion

*Salmonella typhimurium* is a foodborne pathogen with an extremely wide range of transmission. Statistics show that 70% to 80% of foodborne bacterial outbreaks in China are caused by *Salmonella* [25]. Many microorganisms differentiate into biofilm multicellular communities to thrive in harsh and changing environments, a survival strategy considered one of the most widespread and successful life modes on Earth [26]. The special structure of biofilm makes the embedded bacteria 1000–1500 times more drug-resistant than planktonic bacteria [27]. Therefore, developing effective agents to inhibit or disrupt biofilm formation and structure is crucial for controlling pathogenic infections. This remains a critical challenge in medicine and microbiology. Probiotics and their metabolites, widely used in animal production, have replaced chemical additives as potential natural antibacterial agents to extend shelf life and enhance food safety [28]. Many studies have shown that probiotic metabolites strongly inhibit *Salmonella*. Our study found that *B. licheniformis* significantly inhibits *Salmonella typhimurium* growth, prevents biofilm formation, and disrupts mature biofilms. Additionally, *B. licheniformis* exhibits unique regulatory effects on *Salmonella typhimurium* motility and quorum sensing (QS).

MIC is often used to determine the optimal inhibitory dose of a substance [29]. The DIZ can be used to determine whether there is a difference between the antibacterial effect of *Bacillus licheniformis* and that of traditional antibiotics [30]. Our study found that *Bacillus licheniformis* significantly inhibited the growth of *Salmonella typhimurium* within 0–12 h, but the inhibitory effect weakened after 24 h. Tazehabadi et al. (2021) findings showed that in liquid environments, *Bacillus subtilis* KATMIRA1933 and *Bacillus amyloliquefaciens* B-1895 did not significantly inhibit the number of planktonic *Salmonella* at 24 h and 48 h, which is consistent with our results [31]; this aligns with the known bacteriostatic properties of *B. licheniformis*. We hypothesize that this may be due to the binding and consumption of its antibacterial substances by *Salmonella typhimurium* cells.

Biofilms can protect bacteria, increase the resistance of bacteria to antibiotics and host immune responses, and act as a diffusion barrier [32]. Biofilm content was determined via crystal violet staining. Experimental results show that treatment with *B. licheniformis* significantly decreased production of both mature and immature *Salmonella* biofilms. This indicates that *B. licheniformis* can inhibit the formation of *Salmonella typhimurium* biofilms and also exerts a destructive and decomposing effect on mature ones. Same as the results of Zammuto et al. [33], who found that BS B3-15 disrupted the established biofilms of Pseudomonas aeruginosa and Staphylococcus aureus and their early adhesion on polystyrene surfaces.

Generally, extracellular polymeric substances (EPS) are roughly considered to be composed of the mucus secreted by bacteria and the exfoliated substances of certain components on the bacterial surface [34]. EPS serves as the structural and functional scaffold of biofilms and possesses properties such as the ability to adhere to both biotic and abiotic surfaces, resistance to antimicrobial agents, tolerance to environmental changes (such as pH, osmolality, ultraviolet light, etc.), promotion of intercellular communication, and nutrient capture [26,35,36]. This structure also contributes to the mechanical stability of biofilms and is a key factor in the formation, maintenance, and manifestation of the physical and chemical properties of microbial biofilms [34,37]. After the disappearance of microbial populations, the EPS they produce can still persist for a long time [38]. The main components of biofilms include water, extracellular polysaccharides, extracellular proteins, lipids, surfactants, and extracellular DNA (e-DNA), and these substances play a role in the formation of the matrix scaffold and the exertion of functions of biofilms [39,40]. e-DNA is an extremely versatile molecule that represents not only an inert structural element of biofilm structure but also a participant in a variety of tasks [41]. In the case of biofilms, e-DNA has been found to actively promote biofilm tolerance by limiting the diffusion of antimicrobial agents [42,43], can direct biofilm diffusion [44], and act as a source of nutrients during starvation [45]. In this study, by measuring the contents of extracellular polysaccharide, e-DNA, and extracellular protease, our results show that *B. licheniformis* significantly inhibits *Salmonella typhimurium* biofilms and disrupts their structure. Combined with the analysis of growth curve experiment results, when the time exceeds 24 h, *B. licheniformis* has no significant effect on the bacterial count of *Salmonella typhimurium*, but the biofilm components are significantly reduced. It can be inferred that the anti-biofilm activity of *B. licheniformis* is achieved by interfering with biofilm components rather than through growth inhibition.

Biofilm is directly proportional to the number of viable cells [46]. Microscopic observation experiment: after treatment with crystal violet staining, both immature and mature biofilms, following treatment with *B. licheniformis*, showed a significant decrease in the number of micro-colonies and macro-colonies in their optical images. Scanning electron microscopy and confocal fluorescence microscopy show that after treatment with *B. licheniformis*, the number of bacteria in mature/immature biofilms decreased, and the number of dead bacteria increased, which is consistent with the results of crystal violet staining. These experimental results, including microscopic observations and the analysis of biofilm components, corroborate each other, strongly indicating that *B. licheniformis* can not only inhibit the production of extracellular polysaccharides, extracellular proteases, and e-DNA of *Salmonella typhimurium* biofilm components but is also able to decompose these components in mature *Salmonella typhimurium* biofilms, thereby having an important impact on the function of *Salmonella typhimurium* biofilms. Based on the comprehensive analysis of the effects of *B. licheniformis* on the growth and biofilm (components, structure) of *Salmonella typhimurium*, *B. licheniformis* not only inhibits the growth of *Salmonella typhimurium* but also suppresses the formation of biofilms. These findings provide a solid experimental basis for further exploring the application of *B. licheniformis* in the field of antibacterial and anti-biofilm. Given the antibacterial and anti-biofilm properties of *B. licheniformis*, it is expected to become an effective inhibitor for preventing or early-stage removal of *Salmonella typhimurium* biofilms. Combined with its biological safety, it holds promise for application in food processing and other related settings.

Swimming and swarming are the migration patterns of bacteria inside and on the surface of the substrate [47], respectively, and they are criteria for measuring the motility of bacteria. *Salmonella* uses pili or flagella to swim and move in swarms in low-agar environments and on semi-solid surfaces, respectively [48]. Once *Salmonella* adheres to the intestinal wall, it can produce biofilms, indicating that the motility of *Salmonella* is closely related to the formation of biofilms [49,50]. Whether *B. licheniformis* reduces biofilm formation by inhibiting these two activities of *Salmonella typhimurium* is not directly demonstrated by direct experimental results at present. However, our experimental results show that *B. licheniformis* inhibits both *Salmonella typhimurium* swimming and swarming motility and biofilm formation, and both effects are concentration-dependent. This indicates that *B. licheniformis* may inhibit biofilm formation by suppressing the swimming and swarming motility of *Salmonella typhimurium*.

QS is the ability of microorganisms to monitor their population density and control gene expression, including AI release, recognition, and detection. *C. violaceum* ATCC 12472 is a commonly used indicator strain [51,52]. In the present study, *B. licheniformis* significantly reduced the production of violacein in *C. violaceum* ATCC 12472. Consistent with this study, the AHL lactonase (AiiA) expressed by a strain of *B. licheniformis* DAHB1 screened from Fenneropenaeus indicus exhibited broad-spectrum AHL substrate specificity, and its purified recombinant AiiA can inhibit the development of Vibrio biofilms [17].

In *Salmonella*, the activity of SdiA is extremely low at 37 °C in the absence of AHL [53]. This suggests that *B. licheniformis* may promote the overexpression of the *sdiA* gene to regulate other aspects of *Salmonella*. *Salmonella* can produce AI-2 and release it extracellularly. The overexpression of *lsrR* leads to decreased expression levels of SPI-1 and flagellar genes, thereby inhibiting AI-2-mediated quorum sensing [54]. In our experiment, *B. licheniformis* upregulated the expression of *lsrA* and *lsrR* genes in the type II QS system, consequently inhibiting the type II QS system. This finding aligns with the results of *B. licheniformis*’s effects on *Salmonella typhimurium* swarming and swimming motility assays. It indicates that *B. licheniformis* may inhibit the motility of *Salmonella typhimurium* by suppressing the type II QS system.

Many factors may affect *Salmonella typhimurium* attachment/biofilm formation, such as temperature, oxygen levels, dynamic conditions, etc. [55]. *Salmonella typhimurium* is more likely to form biofilms at 30 °C in microaerobic or anaerobic, weakly acidic, and nutrient-limited environments [55,56]. Although our study demonstrated the antibacterial and anti-biofilm activities of *B. licheniformis*, it was only conducted in the laboratory. Further studies are needed to confirm the activity of *B. licheniformis* in other environments. Previous studies have demonstrated that dietary supplementation with *B. licheniformis* significantly improves growth performance, immune indices, and antioxidant enzyme activities in broilers infected with *Clostridium perfringens*, while also alleviating jejunal mucosal damage and reducing the elevation of pro-inflammatory cytokine levels [57]. However, whether *B. licheniformis* can mitigate the inflammatory response and intestinal damage induced by *Salmonella typhimurium* infection remains to be validated through animal experiments.

## 5. Conclusions

In conclusion, this study reveals the significant antibacterial and anti-biofilm potential of *B. licheniformis* against *Salmonella typhimurium*. Specifically, *B. licheniformis* significantly inhibits *Salmonella typhimurium* growth. In terms of biofilms, *B. licheniformis* effectively prevents biofilm formation and disrupts mature biofilms.

This anti-biofilm effect may be mediated by targeting key biofilm components such as EPS, proteases, and e-DNA. Additionally, *B. licheniformis* inhibits *Salmonella typhimurium* motility and quorum sensing (QS), suggesting its biofilm-disrupting activity may involve suppressing motility and QS signaling. Collectively, these findings indicate that *B. licheniformis* represents a promising antibacterial and anti-biofilm agent with broad applications in combating bacterial infections and related diseases.

## Figures and Tables

**Table 1:** List of Q-PCR primers.

Gene Product	Primer Sequence (5′–3′)	Accession Number
*sdiA*	F: CGTACCACTTATCCTCCGGC R: TCTGCGTAATCCGAAACGCT	KF381283.1
*rck*	F: AACGTACTGGTCGCTGATGG R: ATGCTCCTTCACTTCAGCCC	KX807610.1
*luxS*	F: ACGCTTGAGCATCTGTTTGC R: CTGCACTTTCAGCACATCCG	KF381282.1
*lsrA*	F: GTCCCTGCAAGCCAAACAAG R: GGTGTGCGTTGCAGTCATTT	CP099973.1
*lsrK*	F: AAAAGCTAGTGCGCTGGGAT R: GGGATGTCGTTAAGCCACCA	CP099973.1
*LsrR*	F: AAATAGCGTGCGGGATGTGA R: GAATATCGCCTACTGCGCCT	CP099973.1
*16sRNA*	F: CGATGTCTACTTGGAGGTTGTG R: CTCTGGAAAGTTCTGTGGATGTC	NR074910.1

**Table 2:** The diameter of inhibition zone in *Bacillus licheniformis* against *Salmonella typhimurium*.

DIZ (mm)	*B. licheniformis*					Neomycin Sulfate	*p*-Value
	6.25 mg/mL	12.5 mg/mL	25 mg/mL	50 mg/mL	100 mg/mL	100 μg/mL	
***Salmonella*** ***typhimurium***	11.56 ± 1.74 ^e^	13.79 ± 1.37 ^d^	16.26 ± 1.49 ^c^	18.31 ± 1.56 ^b^	19.98 ± 1.38 ^a^	13.43 ± 1.75 ^d^	<0.01

Different lowercase letters between groups indicate significant differences (*p* < 0.05) (the same below).

## Data Availability

The original contributions presented in the study are included in the article, further inquiries can be directed to the corresponding author.
